# Cefdinir and β-Lactamase Inhibitor Independent Efficacy Against *Mycobacterium tuberculosis*

**DOI:** 10.3389/fphar.2021.677005

**Published:** 2021-06-07

**Authors:** Shashikant Srivastava, Tania Thomas, Dave Howe, Lesibana Malinga, Prithvi Raj, Jan-Willem Alffenaar, Tawanda Gumbo

**Affiliations:** ^1^ Department of Pulmonary Immunology, University of Texas Health Science Centre, Tyler, TX, United States; ^2^ Department of Immunology, UT Southwestern Medical Center, Dallas, TX, United States; ^3^ Division of Infectious Diseases and International Health, University of Virginia, Charlottesville, VA, United States; ^4^ Quantitative Preclinical and Clinical Sciences Department, Praedicare Inc., Dallas, TX, United States; ^5^ Department of Internal Medicine, University of Pretoria, Pretoria, South Africa; ^6^ Faculty of Medicine and Health, School of Pharmacy, The University of Sydney, Sydney, NSW, Australia; ^7^ Westmead Hospital, Sydney, NSW, Australia; ^8^ Marie Bashir Institute of Infectious Diseases, The University of Sydney, Sydney, NSW, Australia; ^9^ Department of Medicine, University of Cape Town, Cape Town, South Africa

**Keywords:** cephalosporins, avibactam, hollow fiber model, multi-drug resistance, pharmacokinetics/pharmacodynamics

## Abstract

**Background:** There is renewed interest in repurposing β-lactam antibiotics for treatment of tuberculosis (TB). We investigated efficacy of cefdinir, that withstand the β-lactamase enzyme present in many bacteria, against drug-susceptible and multi-drug resistant (MDR) *Mycobacterium tuberculosis* (*Mtb*).

**Methods:** Minimum inhibitory concentration (MIC) experiments were performed with *Mtb* H37Ra, eight drug-susceptible, and 12 MDR-TB clinical isolates with and without the β-lactamase inhibitor, avibactam at 15 mg/L final concentration. Next, we performed dose-response study with *Mtb* H37Ra in test-tubes followed by a sterilizing activity study in the pre-clinical hollow fiber model of tuberculosis (HFS-TB) study using an MDR-TB clinical strain. Inhibitory sigmoid E_max_ model was used to describe the relationship between the drug exposure and bacterial burden.

**Results:** Cefdinir MIC for *Mtb* H37Ra was 4 and 2 mg/L with or without avibactam, respectively. The MIC of the clinical strains ranged between 0.5 and 16 mg/L. In the test-tube experiments, cefdinir killed 4.93 + 0.07 log_10_ CFU/ml *Mtb* H37Ra in 7 days. In the HFS-TB studies, cefdinir showed dose-dependent killing of MDR-TB, without combination of avibactam. The cefdinir PK/PD index linked to the *Mtb* sterilizing efficacy was identified as the ratio of area under the concentration-time curve to MIC (AUC_0–24_/MIC) and optimal exposure was calculated as AUC_0–24_/MIC of 578.86. There was no resistance emergence to cefdinir in the HFS-TB.

**Conclusion:** In the HFS-TB model, cefdinir showed efficacy against both drug susceptible and MDR-TB without combination of β-lactamase inhibitor. However, clinical validation of these findings remains to be determined.

## Introduction

Bedaquiline and delamanid are the new addition to the anti-TB armament to combat multi-drug resistant tuberculosis (MDR-TB) (Gler et al., 2012; Cox and Laessig, 2014), however, the emergence of drug resistance to these newly developed drugs, designed specifically for *Mycobacterium tuberculosis* (*Mtb*), was quickly reported (Andries et al., 2014; Bloemberg et al., 2015; Hoffmann et al., 2016). Thus, MDR-TB still remains a major global health problem (Dheda et al., 2017) and quest for potent anti-TB drug continues. Antimicrobial drug development is a time consuming and expensive process as well as less lucrative for the pharmaceutical industry compare to the anti-cancer or anti-inflammatory drugs (Cole, 2014). Therefore, repurposing of antibiotics that are already in clinical use appear to be an attractive, fast and pragmatic way to identify drugs with anti-TB activity (Maitra et al., 2015; Ramon-Garcia et al., 2016; Alffenaar et al., 2019). The advantages of repurposing the drugs include availability of post-licensure data regarding dosing and drug safety that makes the repurposed drugs readily available for off-label clinical use.

β-lactams are the most widely used class of antibiotics. Several β-lactams, namely benzyl penicillin, ceftazidime, ceftriaxone, and faropenem, have shown efficacy against *Mtb* using the hollow fiber system model of TB (HFS-TB) (Deshpande et al., 2016; Srivastava et al., 2016a; Deshpande et al., 2017b; Deshpande et al., 2017c; Deshpande et al., 2018; Srivastava et al., 2020a; Gumbo et al., 2021). Since, *Mtb* can be present in different metabolic populations (Mitchison, 1979), it is of interest to continue the screening for a β-lactam that have efficacy against different *Mtb* metabolic populations. Cefdinir is a third-generation oral semi-synthetic cephalosporin used for the treatment of Gram-positive and Gram-negative infections. It binds to the penicillin binding proteins, leading to the damage of the cell wall, cell lysis and ultimately death of drug susceptible bacteria. Cefdinir is also stable to hydrolysis by commonly occurring plasmid-mediated β-lactamases which means that it can potentially resist *Mtb* β-lactamase and could be used without addition of an β-lactamase inhibitor (Gordon et al., 2018). An elsewhere published drug screening study also suggest cefdinir as a potential candidate for evaluation against *Mtb* (Ramon-Garcia et al., 2016).

Cefdinir displays a linear pharmacokinetic profile over the 200–400 mg dose range that changes to nonlinear at higher dose of 600 mg. In adults, single dose of 300 and 600 mg results in mean C_max_ of 1.6 and 2.87 mg/L, respectively. Whereas, in children the oral dose of 7 and 14 mg/kg was observed to achieve C_max_ of 2.3 and 3.86 mg/L, respectively. Cefdinir is 60–73% plasma protein bound, estimated bioavailability is ∼20%, is widely distributed and achieves clinically relevant concentrations in the epithelial lining fluid. (Perry and Scott, 2004). Thus, theoretically cefdinir has the potential to be used for the treatment of pulmonary disease caused by *Mtb*.

Therefore, the aim of our study was to perform pharmacokinetic/pharmacodynamics (PK/PD) studies of cefdinir, against two different metabolic populations of *Mtb*, using the pre-clinical HFS-TB model of bactericidal and sterilizing effect (Gumbo et al., 2009; Srivastava et al., 2016b) to determine the PK/PD optimized exposure target of cefdinir for treatment of TB.

## Methods

### Bacterial Strains, Drugs, and Supplies

We used the *Mtb* laboratory strain H37Ra (ATCC#25177) and 20 clinical isolates (eight drug susceptible and 12 MDR-TB) provided by the South African Medical Research Council, TB Platform. The ethical approval was obtained from the Human Research Ethics Committee (UPHREC) at the Faculty of Health Sciences, University of Pretoria (Ethics Reference Number: 239/2016). Storage and culture conditions for log-phase growth *Mtb* cultures and transformation into semi-dormant bacteria for sterilizing activity experiments were as described in our previous publications (Gumbo et al., 2009; Srivastava et al., 2011a). Cefdinir was purchased from Sigma Aldrich (St Louis, MO, United States), and β-lactamase inhibitor, avibactam, was synthesized by the BOC Sciences (Shirley, NY, United States). Hollow fiber cartridges were purchased from FiberCell (Fredrick, MD, United States). BD BACTEC^TM^ MGIT^TM^ automated mycobacterial detection system and supplies were purchased from Becton, Dickinson and Company (NJ, United States).

### Cefdinir Minimum Inhibitory Concentration

We used two different methods to determine the cefdinir MIC—broth micro-dilution (CLSI, 2018), and MGIT liquid culture method (Bastian et al., 2001; Rusch-Gerdes et al., 2006; Deshpande et al., 2017b). The inoculum was prepared using the log-phase growth culture of *Mtb* H37Ra or the clinical isolates. The drug concentration range was 0, 1, 2, 4, 8, 16, 32, and 64 mg/L with or without combination of avibactam at a concentration of 15 mg/L, based on our previous experiments (Deshpande et al., 2017b; Srivastava et al., 2020b). Five hundred microlitres of the inoculum was added to each MGIT tube supplemented with 900 μl oleic acid, albumin, dextrose, and catalase [OADC] enrichment and 100 μl the drug; thus, a total volume of 8.5 ml. The MGIT time to positive (TTP) was recorded using the EpiCenter software (Bastian et al., 2001; Rusch-Gerdes et al., 2006). For the broth-micro dilution method, the inoculum preparation and the drug concentrations were the same as for the MGIT method, except the experiment was performed in 96-well plates. After 7 days of incubation, plates were visually examined using an inverted mirror and the concentration showing complete inhibition of the bacterial growth was recorded as the MIC. The experiments were performed twice with two replicates for each concentration.

### Cefdinir Concentration-Response at Static Concentration in Test-Tubes

The preparation of the inoculum and cefdinir concentration range were same as described above, except the experiment was carried out in 15 ml screw caped tubes with a total volume of 5 ml. The log-phase growth *Mtb* H37Ra cultures were co-incubated with different drug concentration, in replicate of three, at 37°C under 5% CO_2_ and shaking conditions for 7 days. On day 7, the cultures were washed twice with normal saline to remove the carry-over drug, serially 10-fold diluted in normal saline and inoculated on Middlebrook 7H10 agar supplemented with 10% OADC (herein termed “agar”). The colony forming unit (CFU) with each concentration were recorded after 21 days of incubation at 37 C under 5% CO_2_. The four-parameter inhibitory sigmoid E_max_ model was used to determine the relationship between the drug concentration and the bacterial burden.

### Cefdinir Bactericidal Activity With or Without the β-Lactamase Inhibitor in the Hollow Fiber Model of Tuberculosis

In the preliminary drug screening studies (at static concentration), we found that there was no significant difference (data not shown) in cefdinir *Mtb* killing with or without addition of the β-lactamase inhibitor, avibactam. To confirm this, we performed an experiment with log-phase growth *Mtb* cultures to determine if the effect persists at the dynamic or fluctuating concentrations using the HFS-TB model of bactericidal effect (Srivastava et al., 2011a; Srivastava et al., 2011b). Twenty mL of log-phase growth *Mtb* H37Ra culture were inoculated into the peripheral compartment of each of eight HFS-TB units. Since percent of the time drug concentration persist above MIC (%T_MIC_) is the PK/PD index linked to the β-lactam’s efficacy, the HFS-TB were treated with different cefdinir doses to achieve 50, 75, and 100%T_MIC_ with or without combination of avibactam at concentration of 15 mg/L. Drugs were infused into the central compartment via a computerized syringe pump over 1 h. The fresh media inflow rate (i.e., dilution) was set to mimic a 2 h cefdinir half-life (Zhang et al., 2011). The central compartment of each of the eight HFS-TB unit was sampled before drug infusion followed by at 1, 2, 3, 6, 12, 15, 18, 23.5, 25, 26, 28, 30, 36, 42, and 47.5 h after the administration of the first dose to validate the drug concentration-time profile. The peripheral compartment of each HFS-TB unit was sampled on day 0, 3, 7, 10, 14, 21, and 28 to quantify the bacterial burden. The samples were washed twice with normal saline to remove any carry over drug, serially 10-fold diluted and cultured on agar. The processed samples were also inoculated on agar supplemented with three times the cefdinir MIC to determine the proportion of the cefdinir resistant *Mtb* sub-population. The intent was not to determine the absolute change in the MIC. The cultures were incubated at 37°C for 21 days before CFUs were recorded. One portion of the processed sample (undiluted) was also inoculated in the MGIT tubes to record the TTP, as second pharmacodynamic measurement.

### Cefdinir Sterilizing Activity Against MDR-TB in the Hollow Fiber Model of Tuberculosis

It is important that a drug can kill different metabolic populations of *Mtb* (Mitchison, 1979). Also, the efficacy determined using standard drug-suscetible laboratory strain may not be same against MDR-TB strains. Therefore, we performed sterilizing activity studies of cefdinir using a MDR-TB clinical strain (16D). The isoniazid and rifampin phenotypic resistance in this clinical starin was also confirmed by whole genome sequencing, using the methods described previously (Srivastava et al., 2006; Deshpande et al., 2017b; Srivastava et al., 2017). The whole genome sequencing showed presence of drug resistance associated mutation in *katG* (Ser315Thr), *rpoB* (Ser450Leu, Tyr564His)*, embB* (Met306Val)*, pncA* (Val139Gly)*, gidB* (Leu16Arg, Ser100Phe) and *gyrA* (Clu21Gln, Ser95Thr, Gly247Ser, Gly668Asp) and ponA1 (Pro631Ser) genes of *Mtb*.

Prior to the experiment, 4 day old log-phase growth cultures were transformed into semi-dormant bacilli (SDB) growing under acidic condition (pH 5.8). The detailed method of transformation to SDB has been published elsewhere (Gumbo et al., 2009; Srivastava et al., 2011a). Next, we examined 14 different doses of cefdinir, in a combined dose-effect and dose-frationation study design to achieve 0, 8, 16, 25, 32, 42, 50, 64, 84, and 100%T_MIC_ with either once daily or twice daily dosing schedule. The number of HFS-TB units was 16, including two non-treated control systems. The study was performed without addition of avibactam. The sampling of the peripheral compartment to validate the drug concentration-time profile and of the central compartment to determine the total as well as the drug resistant sub-population was performed as described above.

### Drug Concentration Measurements

Avibactam was measured using a previously validated method (Deshpande et al., 2017a; Deshpande et al., 2017b; Srivastava et al., 2020b). We developed an LC-MS/MS methods for measurement of cefdinir. Briefly, Cefdinir and ceftazidime-d5 (internal standard, IS) were purchased from Sigma (St Louis, MO, United States) and Toronto Research Chemicals (Toronto, Canada), respectively. LC-MS/MS analysis was performed using Waters Acquity UPLC coupled with Waters Xevo TQ mass spectrometer. Data was collected using MassLynx version 4.1 SCN810 software. Separation was achieved by injecting 2 μl of sample on a Waters Acquity UPLC HSS T3 column (50 × 2.1 mm; 1.8 μm) using a binary gradient. Stock solutions of the standard and IS were prepared in 80:20 methanol:water at a concentration of 1 mg/ml. Calibration curve, low- and high-quality control samples (LQC and HQC) were prepared by diluting the stock solution in blank medium. Samples were diluted 1:20 with IS solution in 0.1% aqueous formic acid (FA). Solvents for UPLC were: (A) 0.1% aqueous FA, and (B) 0.1% FA in methanol. Flow rate was 0.2 ml/min; total run time was 6 min. Compounds were detected using positive ESI in MRM mode. The transitions used were *m/z* 396–227 (cefdinir), and *m/z* 552–468 (ceftazidime-d5). The between day percentage coefficient of variation (%CV) for analysis of low and high (brackets) quality controls were 3% (1%). The inter- and intra-day %CV were 4 and 2%. The lower limit of quantitation was 0.01 µg/ml.

### Data Analysis

We performed pharmacokinetic modeling, with priors from literature, using the measured drug concentrations in the HFS-TB (Shimada et al., 1989; Srivastava et al., 2011a; Srivastava et al., 2017). We used two different software’s, ADAPT (D'argenio and Schumitzky, 1997) and Phoenix WinNonlin 8.1 (Certara USA, Inc., MO, United States) for pharmacokinetic modeling to compare the results. The measured drug concentration in each HFS-TB unit was used to calculate the ratio of peak to MIC (C_max_/MIC), 0–24 h area under the concentration-time curve to MIC (AUC_0–24_/MIC), and the %T_MIC_ for each cefdinir doses. Drug concentration and bacterial response relationships were examined using the inhibitory sigmoid E_max_ model for microbial kill, and one-way analysis of variance to compare the dosing schedule was performed in GraphPad Prism v 8.0 (La Jolla, CA, United States).

## Results

**FIGURE 1:**
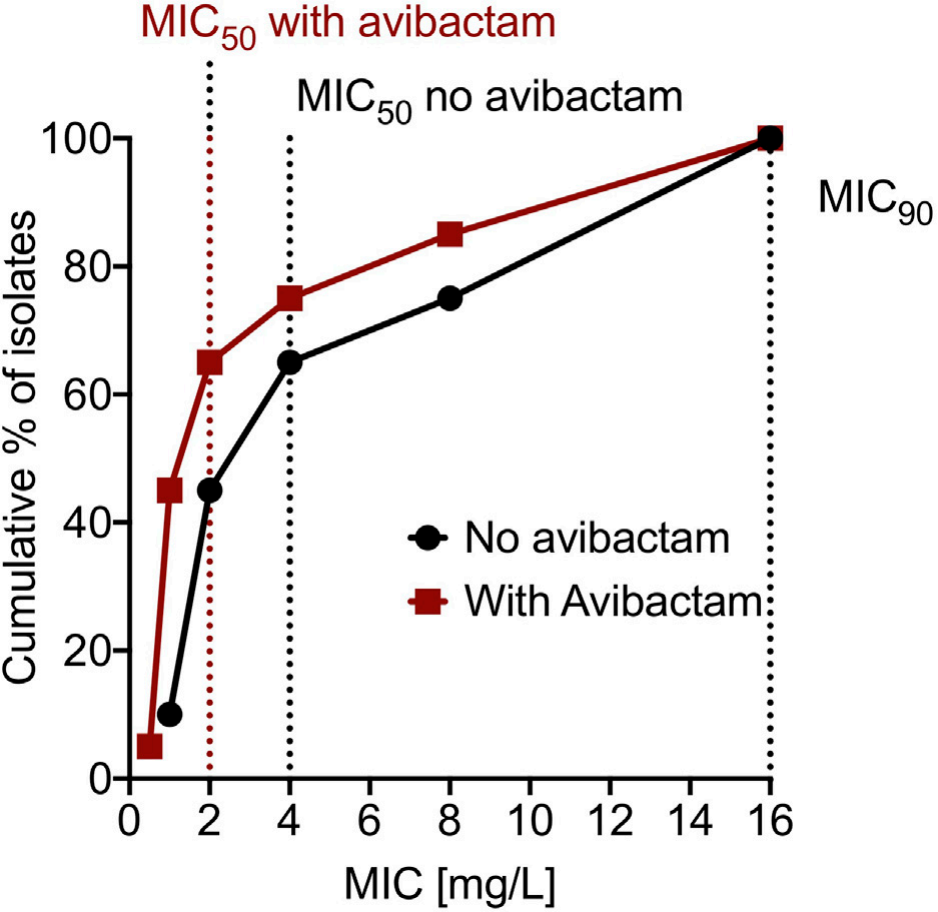
Cumulative percentage of clinical isolates at different MIC. The dotted line on the x-axis represent MIC_50_ and MIC_90_ for the 20 clinical isolates.

**FIGURE 2:**
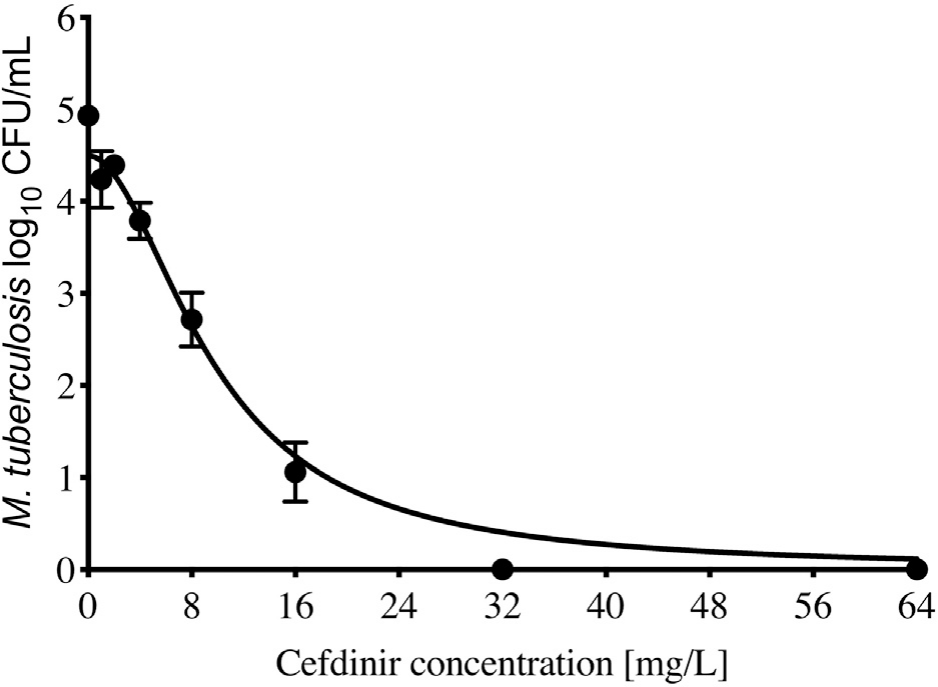
Cefdinir efficacy against *M. tuberculosis*. Compare to the non-treated controls, cefdinir alone at a concentration of 32 mg/L killed 4.93 + 0.07 log_10_ CFU/ml log-phase growth *Mtb* in 7 days static concentration experiment.

The cefdinir MIC of the laboratory strain H37Ra was 4 and 2 mg/L with and without 15 mg/L avibactam, respectively. The MIC of the MDR-TB clinical isolate (16D) used in the subsequent HFS-TB study was 1 mg/L with or without avibactam, by both MGIT and the broth dilution method. Table 1 show the MIC distribution of cefdinir among the clinical isolates, whereas Figure 1 show the cumulative percentage of isolates at each MIC concentration. Figure 2 describes the results of the cefdinir concentration response study, performed in test-tubes at static concentration*,* where the effective concentration associated with 50% of the bacterial kill (EC_50_) was calculated as 9.64 mg/L with hill coefficient (H) as 1.93 and an *r* ^2^ of 0.97.

**TABLE 1:** Cefdinir MIC of drug susceptible and MDR-TB isolates with or with combination of avibactam at 15 mg/L.

Isolate	Rifampin (1 mg/L)	Isoniazid(0.1 mg/L)	Cefdinir	Cefdinir + Avibactam
1A	S	S	4	2
3A	S	S	2	2
6B	S	S	2	1
8A	S	S	4	4
11B	S	S	2	1
14A	S	S	8	2
16A	S	S	1	0.5
18B	S	S	16	8
1C1	R	R	8	1
3D3	R	R	4	2
5D	R	R	2	1
6C	R	R	16	16
7C4	R	R	16	16
8C	R	R	16	8
10C2	R	R	4	4
11D1	R	R	16	16
16D	R	R	1	1
17D3	R	R	2	1
19C4	R	R	2	1
20D2	R	R	2	1
MIC_50_			4	2
MIC_90_			16	16

### β-Lactamase Inhibitor Independent Bactericidal Activity in the Hollow Fiber Model of Tuberculosis

**FIGURE 3:**
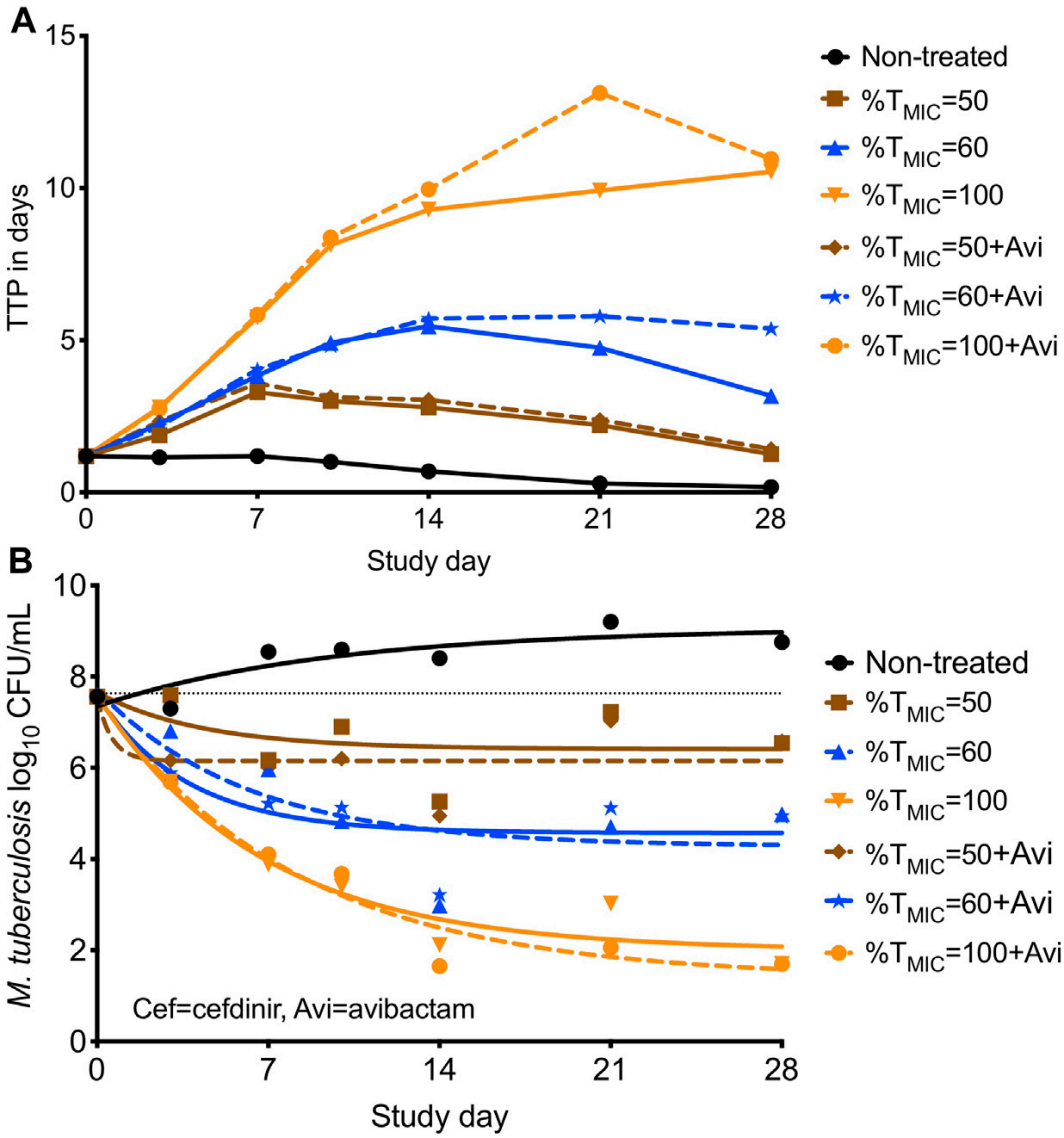
Cefdinir’s bactericidal activity against *M. tuberculosis* with or without avibactam in the hollow fiber system. **(A)** Higher the TTP, lower the bacterial burden. The TTP in the HFS-TB treated with different cefdinir exposures [%T_MIC_] were virtually similar, irrespective of the presence of absence of avibactam, **(B)** The CFU/mL results were similar to that of the TTP. Combination of avibactam did not result in improved bacterial kill. *Avi, avibactam.

Since the circulating media in the HFS-TB was Middlebrook 7H9 both with 10% dextrose (i.e., no protein present binding), the measured drug concentrations represent the free or available drug in the HFS-TB units. The calculated C_max_/MIC with three cefdinir doses were 4.46, 16.34, 82.88; %T_MIC_ were calculated as 50, 60, and 100; and the corresponding AUC_0–24_/MIC were 31.16, 98.17, and 454.7. As shown in Figure 3A, difference in the TTP, recorded in the HFS-TB units treated with different cefdinir exposures in the presence or absence of avibactam was not statistically significant (*p* > 0.05). The kill curves with each cefdinir exposure, with or without avibactam, shown in Figure 3B, was not significantly different, similar to the TTP results. All three cefdinir exposures killed drug susceptible *Mtb* H37Ra, though the extents of kill varied in a dose dependent manner (2.11 vs 3.67 vs 6.95 log_10_ CFU/ml, respectively in 28 days).

### Cefdinir Sterilizing Activity in the Hollow Fiber Model of Tuberculosis

**FIGURE 4:**
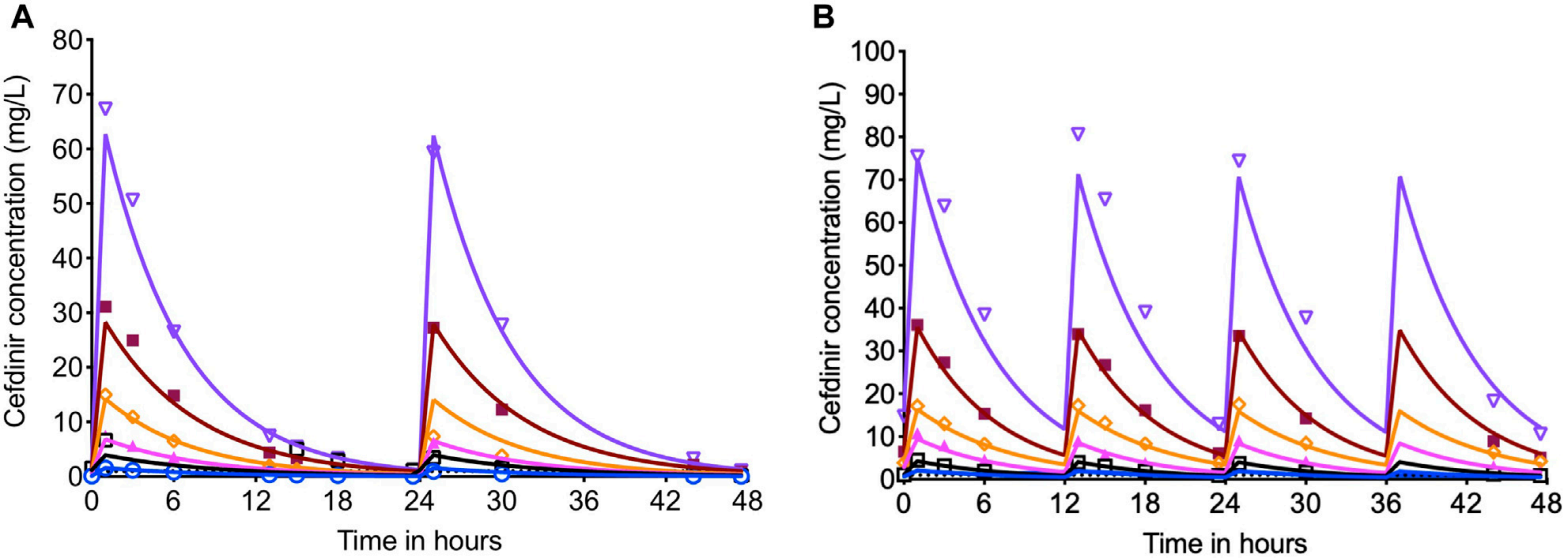
Cefdinir concentration-time profile in the HFS-TB. The cefdinir doses were selected to achieve different %T_MIC_. **(A)** The concentration time profile of cefdinir with once daily or **(B)** twice daily dosing schedule. The solid lines represent modeled concentrations, and the symbols represent the observed concentrations in the HFS-TB.

Since in the HFS-TB study performed with the log-phase growth cultures there was no significant different in the bacterial burden in the systems treated with or without combination of avibactam, this set of experiment was performed with cefdinir alone. Figures 4A,B show the PK modeled predicted and observed cefdinir concentrations in each HFS-TB unit, treated with once or twice daily dosing schedule. In the HFS-TB, the cefdinir clearance was calculated as 0.368 (95% CI: 0.366–0.369) L/h, volume of distribution of 0.248 (95% CI: 0.229–0.267) L, and half-life of 4.679 (95% CI: 4.33–5.038) h.

**FIGURE 5:**
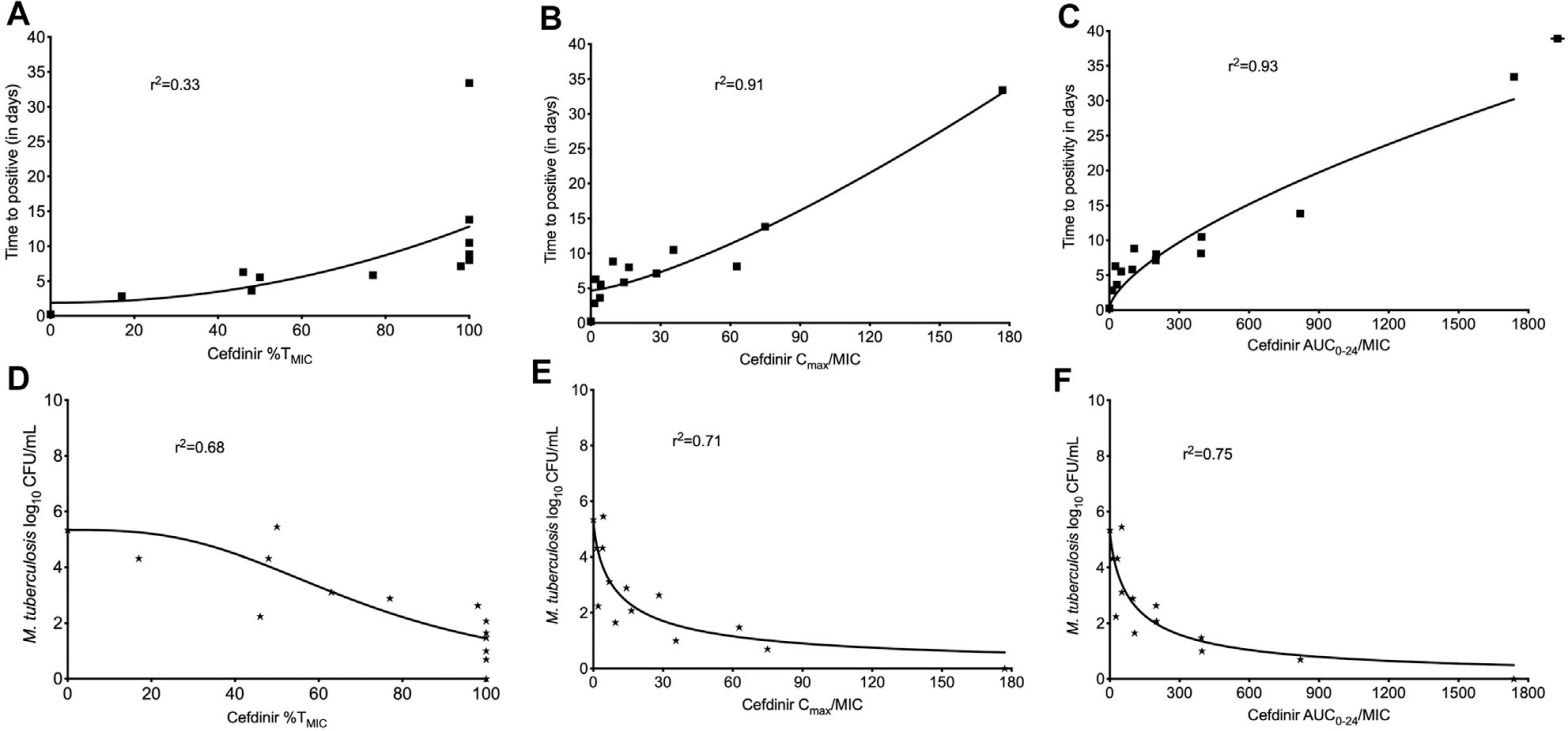
Cefdinir sterilizing activity against multi-drug resistant tuberculosis. The cefdinir doses were selected to mimic different %TMIC. However, the results of TTP, as a surrogate of bacterial burden, were plotted against the **(A)** %T_MIC_, **(B)** C_max_/MIC, and **(C)** AUC_0–24_/MIC. On day 28, the ratio of AUC_0–24_/MIC best described the cefdinir *Mtb* kill. Similarly, the relationship between the bacterial burden as log_10_ CFU/ml and **(D)** %T_MIC_, **(E)** C_max_/MIC, and **(F)** AUC_0–24_/MIC on study day 28. Based on the lowest AIC score, AUC_0–24_/MIC was selected as the PK/PD index for cefdinir efficacy against *Mtb*.

The extent of MDR-TB bacterial kill with different cefdinir exposure varied in a dose dependent manner. On day 28, the maximal kill (E_max_) compare to the non-treated control with cefdinir exposure of 100%T_MIC_ exposure was 4.02 log_10_ CFU/ml. There was no difference in the bacterial burden in systems treated with cefdinir once daily or twice daily, for the same AUC_0–24_/MIC exposure (*p* > 0.05). Next, we determined the relationship between the drug exposure (C_max_/MIC, AUC_0–24_/MIC or %T_MIC_) and bacterial burden at each sampling time-point using the inhibitory Sigmoid E_max_ model. The Akaike Information Criteria score (AIC) (Akaike, 1974) was used to select the PK/PD parameter (with lowest AIC score) associated with cefdinir microbial kill. Table 2 show the AIC scores for TTP and CFU/ml on each sampling day for each PK/PD index. We found that, on study day 28, for both TTP (Figures 5A–C) and log_10_ CFU/ml (Figures 5D–F), AUC_0-24_/MIC had lower AIC score compare to %T_MIC_ or C_max_/MIC. Therefore, we determined that in the HFS-TB, AUC_0–24_/MIC was the PK/PD index linked to the cefdinir’s sterilizing efficacy against *Mtb*. Using the CFU/ml readouts, the EC_50_ was calculated as an AUC_0–24_/MIC of 104.1 with an H of 0.80. The EC_80_ was calculated an AUC_0–24_/MIC of 578.86. There was no cefdinir resistance recorded on agar supplemented with three times MIC.

**TABLE 2:** Determination of the pharmacokinetic/pharmacodynamic index associated with cefdinir *M. tuberculosis* kill in the HFS-TB. The table show the AIC score for both TTP and CFU readouts. The AUC_0–24_/MIC consistently showed lowest AIC score at each sampling time-point, therefore, selected as the PK/PD index linked to cefdinir efficacy in the HFS-TB.

Study Day	day 3	day 7	day 10	day 14	day 21	day 28
**Time to Positive (TTP)**						
%T_MIC_	5.72	13.18	8.73	29.22	55.31	56.90
C_max_/MIC	0.61	1.079	1.11	1.727	3.67	2.44
AUC_0-24_/MIC	0.59	0.84	1.14	1.61	3.48	2.22
**log_10_ CFU/mL**						
%T_MIC_	2.89	−17.4	0.58	−3.12	8.82	6.03
C_max_/MIC	^a^	−16.26	−1.079	−6.36	6.58	4.88
AUC_0-24_/MIC	-10.05	−17.96	−1.709	−8.57	6.48	2.96

a Not Converged.

## Discussion

Cefdinir is commonly used in the treatment of many community-acquired respiratory tract pathogens, namely *Haemophilus influenzae*, *Moraxella catarrhalis* and penicillin-susceptible *Streptococcus pneumoniae*, and is stable to hydrolysis by commonly occurring plasmid-mediated β-lactamases. Until recently cephalosporins with broad spectrum antibacterial activities were not explored for activity against *Mtb* (Ramon-Garcia et al., 2016; Srivastava et al., 2020c). In the present study, first, we found that cefdinir MIC of the standard laboratory strain, drug susceptible and MDR-TB strains was not affected by the combination of avibactam. These findings are similar to those reported earlier by others (Ramon-Garcia et al., 2016) using different reference and clinical strains of *Mtb*. Likewise, in the bactericidal activity HFS-TB study, where avibactam was used at a constant concentration of 15 mg/L, avibactam combination did not improved mycobacterial kill with different cefdinir exposures. Second, our pre-clinical HFS-TB study show that the ratio of AUC_0–24_/MIC is the PK/PD index linked to the cefdinir efficacy against *Mtb*. We were unable to find cefdinir PK/PD study with *Mtb* as well as reports on cefdinir affecting the pharmacokinetics of co-administered anti-TB drugs. However, there are some reports showing favorable drug interaction profile with other concomitantly administered drugs (Ueno et al., 1993).

Our study has limitation. While we show the cefdinir’s bactericidal and sterilizing efficacy against *Mtb* in the HFS-TB model, we did not performed experiments with intracellular *Mtb*, a subpopulation for which the optimal exposure for kill and resistance suppression remains unknown. Further, we did not perform the analysis for the probability of target attainment with cefdinir clinical doses due to the following reasons. Cefdinir 600 mg achieves an AUC of 11.1 ± 3.87 mg*h/L, whereas the EC_80_ or the optimal exposure of cefdinir in the HFS-TB experiments was determined as an AUC_0–24_/MIC of 578.86. Thus, with the currently recommended clinical dose, the optimal exposure for *Mtb* kill cannot be achieved. Moreover, cefdinir serum to lung tissue penetration ratio is about 31 ± 18%, and to the epithelial lining fluid is 35 ± 83% (Food and Drug Administration, 2007), that means even lower drug exposure at the site of infection, with currently prescribed dose. However, there is one study reporting efficacy of cephalosporins’ including cefdinir, and synergistically enhancing the anti-TB activity of first- and second-line anti-TB drugs as well as with a number of new drugs namely pretomanid, bedaquiline, delamanid, and SQ109 (Ramon-Garcia et al., 2016), in the static concentration experiment. Thus, drug-combination PK/PD studies to determine if cefdinir at currently recommended dose could improve the efficacy of the first- and second-line anti-TB drugs, need to be performed.

To summarize, cefdinir, without combination of β-lactamase inhibitor, has both bactericidal and sterilizing activity against *Mtb*. Availability of oral formulations, penetration into clinically relevant anatomical sites, and efficacy against drug susceptible or MDR-TB strains in absence of a β-lactamase inhibitor make cefdinir an attractive candidate to develop for treatment of TB.

## Data Availability

Upon reasonable request, the raw data supporting the conclusions of this article will be made available by the authors, without undue reservation following institutional policies on data sharing.
