# Erratum: Cefdinir and *β*-Lactamase Inhibitor Independent Efficacy Against *Mycobacterium tuberculosis*

**DOI:** 10.3389/fphar.2022.849039

**Published:** 2022-01-27

**Authors:** Frontiers Production Office

**Affiliations:** Frontiers Media SA, Lausanne, Switzerland

**Keywords:** cephalosporins, avibactam, hollow fiber model, multi-drug resistance, pharmacokinetics/pharmacodynamics

Due to production error, an existing conflict of interest between one of the reviewers and the authors was not declared. The updated Conflict of Interest statement has been included below.

The publisher apologizes for this mistake. The original version of this article has been updated.

